# Anti-leucine-rich Glioma-inactivated-1 Encephalitis with Faciobrachial Dystonic Seizures and Behavioral Problems: A Case Report

**DOI:** 10.31729/jnma.5008

**Published:** 2020-07-31

**Authors:** Ayush Adhikari, Ram Chandra Subedi, Subi Acharya, Raju Paudei

**Affiliations:** 1 Department of Medicine, Shree Birendra Hospital, Chhauni, Kathmandu, Nepal

**Keywords:** *autoimmune*, *case report*, *limbic*, *seizure*

## Abstract

Anti-Leucine-rich glioma-inactivated-1 encephalitis is a subtype of autoimmune encephalitis characterized by cognitive impairment, faciobrachial dystonic seizures, and behavioural changes. There is no reported case from Nepal. We report a case of a 54-year old male who presented with abnormal body movement, behavioural changes, rapid eye movement sleep behavioural changes, and cognitive decline. Investigations revealed magnetic resonance imaging findings of fluid-attenuated inversion recovery intensity signal in bilateral temporal lobes with anti-leucine-rich glioma-inactivated-1 antibody in serum leading to diagnosis of anti-leucine-rich glioma-inactivated-1 encephalitis. He was treated with steroids and intravenous immunoglobulin. Timely diagnosis is vital to prevent complications and improve outcomes.

## INTRODUCTION

Limbic encephalitis, a form of autoimmune encephalitis is common above the age of 45 years and presents with confusion, behavioural changes, seizures and inability to form new memories.^1^ Anti-leucine rich glioma inactivated-1 (anti-LGIl) encephalitis has an incidence of around 0.83 per 100 million and is characterized by rapidly progressive dementia, behavioural problems, faciobrachial dystonic seizures and hyponatremia.^2,3^ Lymphocytic pleocytosis in Cerebrospinal fluid (CSF) and Magnetic resonance imaging (MRI) findings of hyperintense signal on T2-weighted fluid-attenuated inversion recovery sequences are present. Treatment includes steroids, intravenous immunoglobulins, immunosuppressive agents like rituximab and cyclophosphamide while antiepileptic drugs are used to control seizures.^4,5^

## CASE REPORT

A 54-year old male who was evaluated for three episodes of complex partial seizures 2 months back manifesting as perioral automatism with staring look followed by an abnormal jerky movement of extremities lasting few minutes with postictal confusion. He was initially investigated and found to have hyperintensity signal in the left mesial temporal region with a normal Electroencephalogram (EEG) and was started on oxcarbamazepine which was gradually increased to 1200 mg/day. On follow up he presented with cognitive changes with the affection of short-term memory, Montreal cognitive assessment (MOCA) score of 21/30 with irrelevant talks, muttering to self, disturbed sleep with frequent vivid acting in the dreams. He had frequent episodes of short-lasting jerky movements of arm, head, and neck. He intermittently used to have staring episodes with unresponsiveness and confusion. His dosage of oxcarbamazepine was increased to 1800 mg/day and levetiracetam was added to the dosage of 2000 mg/day.

Since the patient continued to have cognitive decline and persisting frequent episodes of faciobrachial seizures, he was admitted for further evaluation. After admission, neuropsychiatric consultation was done for behavioural abnormalities and antipsychotic medication was added. EEG was done which revealed Interictal epileptiform discharges (lEDs) from the bilateral temporal region. The patient's condition deteriorated with the patient being unable to remember his family members and surroundings. He started forgetting if he had eaten or gone to the toilet. Because of the deteriorating behavioural condition and cognitive decline of the patient, differentials were broadened to include autoimmune encephalitis, Creutzfeldt-Jakob disease, and viral encephalitis. Repeat MRI head (Epilepsy Protocol) was advised, CSF examination done and autoimmune antibody panel was sent. Pulse intravenous methylprednisolone 1000 mg/day was also started considering the possibility of autoimmune encephalitis. There was some improvement of his abnormal jerky movements but he continued to have agitation and insomnia with muttering to self.

**Figure 1.**
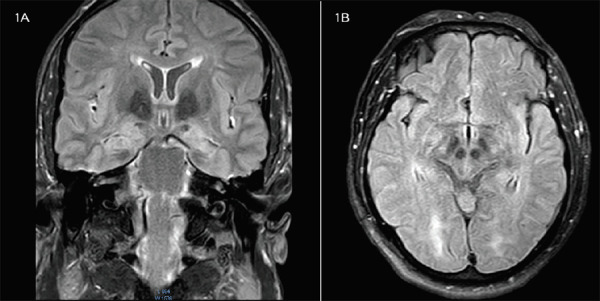
A, B. MRI showing increased FLAIR intensity signal in the bilateral hippocampus with atrophy of the left hippocampus and temporal lobes and insular cortex (A) Coronal view (B) Axial view.

MRI findings in FLAIR (fluid-attenuated inversion recovery) sequence showed hyperintensity signal in bilateral temporal lobes predominantly involving hippocampus and bilateral insular cortex consistent with the strong possibility of autoimmune encephalitis (Figure 1 A, B).

Lumbar puncture reports showed acellular picture with normal protein and glucose with polymerase chain reaction (PCR) for neurotropic viruses (Herpes Simplex virus, Epstein-Barr virus, and Japanese Encephalitis virus) negative. He was also evaluated for occult malignancy doing contrast-enhanced computed tomography (CECT) abdomen and chest which was unremarkable. Thyroid function test was normal with a normal level of anti-thyroid peroxidase antibody (anti-TPO antibody). Routine hematological and biochemical tests were normal. Venereal disease research laboratory (VDRL) test and human immunodeficiency virus (HIV) serology were noncontributory. Subsequently, anti-LGI1 encephalitis, a form of limbic encephalitis was confirmed as an autoimmune encephalitis panel revealed anti-LGI1 antibody positive result in serum. The patient was then shifted to the intensive care unit and intravenous immunoglobulin (IV Ig) was initiated. He received IV Ig 30 gm/day for a total of 5 days. The patient improved dramatically as his faciobrachial seizures subsided and agitation with insomnia and rapid eye movement (REM) behavioural problems also improved. During the hospital stay, the patient also developed hyponatremia recorded lowest upto 124 mEq/L which was managed with restricting the fluid intake as a proposed mechanism is due to syndrome of inappropriate antidiuretic hormone secretion. At the time of discharge, the patient was well oriented, co-operative without episodes of seizure along with normal behaviour and sleeping pattern. However, he still had lapses of short-term memory with MOCA score of 25/30. He was discharged on antiepileptic oxcarbamazepine 1200 mg/day, levetiracetam 2000 mg/day, and clobazam 10 mg/day and tapering dosage of oral steroid.

He was doing fine in regular two weeks follow up when he developed morbilliform erythematous rashes in his body trunk and extremities initially which gradually coalesced with peeling of superficial skin especially of palms and soles. Dermatology consultation was done and the possibility of drug-induced erythroderma was considered. Oxcarbazepine was stopped while other antiepileptic drugs (AED) were continued. His erythroderma gradually improved over the next 1 month and since he was not having further episodes of seizures, another AED was also gradually tapered off and stopped. On subsequent follow-up after 3 months, the patient remained seizure free with normal behavioural activity and sleep pattern though he continued to have lapse in short term memory.

## DISCUSSION

Anti-LGI1 encephalitis is characterized by cognitive impairment, facial-brachial dystonic seizures, sleep disorders, behavioural changes, and is seen in older people with an average age of onset being 63 years.^1,6,7^ Our patient was of age 54 years and presented with muttering to self, vacant staring, abnormal body movements, and sleep disturbances. He developed faciobrachial dystonic seizures while being admitted to the hospital.

The mechanism by which LGI1 antibody causes seizure is not known but it is postulated that LGI1 antibodies were found to disrupt ligand-receptor interaction of LGI1 with ADAM22/23 complex which results in a reversible reduction in synaptic AMPA receptors. When lacking in LGI1, there is a reduction in α-amino-3-hydroxy-5-methyl-4-isoxazolepropionic acid (AMPA) receptor-mediated synaptic transmission and this has been shown in mice models to lead to a seizure.^8^ The types of seizures can vary ranging from focal seizure without loss of consciousness to focal seizure with dyscognitive features, generalized tonic-clonic seizures (GTCS), myoclonic seizure but characteristic seizure for LGI1 antibody is faciobrachial dystonic seizures (FBDS) likely due to affection of basal ganglia circuitry. Among the patients with positive LGI1 antibody, approximately 20%-40% have FBDS, which are brief, frequent, upper limb spasm with the involvement of the ipsilateral face and leg.^9^

The patient was confused and drowsy most of the time, while other times he used to stay up all night speaking irrelevantly. His recent memory was affected while the long term memory was relatively preserved. Hyponatremia, a typical feature of anti-LGI1 encephalitis, was seen during hospital stay which was managed with restricting the fluid as a proposed mechanism of hyponatremia may be due to improper secretion of antidiuretic hormone, related to the simultaneous LGI1 expressions of the hypothalamus and kidney.^1^

The subtle clue to the diagnosis of autoimmune encephalitis has to be kept in mind as these types of encephalitis go unnoticed and mostly managed by psychiatrist thinking as a mere behavioural issue and have a long latent period before diagnosis. Initial presentation of the patient just had complex partial seizure and hyperintensity in MRI head was restricted to left side only so was initially managed as possible Mesial temporal sclerosis (MTLS) but the cognitive decline and faciobrachial seizures on follow up made us think of the strong possibility of autoimmune encephalitis.

MRI findings of anti-LGI1 encephalitis generally show abnormal FLAIR intensity in the hippocampus and medial temporal lobe which may be unilateral or bilateral while CSF findings are mostly normal with some cases showing oligoclonal bands.^2,10,11^ The illustrated case showed FLAIR intensity signal in bilateral temporal lobes with signal changes in the bilateral insular cortex. CSF findings of our patient showed no abnormalities. The clinical features along with MRI findings of FLAIR intensity signals in bilateral temporal lobes and positive LGI1 antibody in serum lead to the diagnosis of anti-LGI1 encephalitis in our patient. Although meso-temporal sclerosis has been described in 25-50% of follow-up MRIs in patients with anti-LGI1 encephalitis, only a few develop epilepsy after resolved encephalitis.^4^

Treatment options include glucocorticoids, intravenous immunoglobulins, mycophenolate mofetil, and plasma exchange.^4^ Rituximab may be an alternative therapy as these seem to be well tolerated in an older population.^5^ After positive MRI findings suggestive of limbic encephalitis, our patient was started on high dose corticosteroids; and intravenous immunoglobulin was initiated after the antibody panel revealed LGI1 antibody in the serum. The patient improved dramatically over the following days and remained seizure-free.

After acute treatment of seizures with antiepileptic medications, the long-term use of antiepileptic medication is controversial and most of the studies show that seizures are poorly controlled with an even higher dosage of individual antiepileptic medication and sometimes even with the combination of antiepileptic medications like in our case. Dystonic seizures were refractory to the antiepileptic medications prescribed in our patient and seizures improved only after starting immunosuppressive and immunomodulation, steroid, and immunoglobulin in our case. This has been the consistent observations in such cases of anti LGI1 antibody-related encephalitis by other studies and authors. Chronic AED use does not appear to be necessary for most autoimmune encephalitis (AIE) patients on long term across separate subtypes of AIE.^12^ After 2 months, the patient developed rash as a side effect of oxcarbazepine which has been seen in other reported cases as well.^8,13^ The rashes subsided with the removal of the drug. Previous studies have shown one-third of patients with LGI1 antibodies treated with carbamazepine had a rash especially in patients with specific pro-immunogenic human leukocyte antigen (HLA) types (HLA DR7 and DRB4).^14,15^ We have not analyzed the HLA haplotype of our patient but the association of rash while taking oxcarbazepine and its improvement after the stoppage of medication strongly suggests the connection of rash with the medication. This case highlights the importance of considering autoimmune encephalitis in cases presenting with atypical seizures along with other cognitive and behavioral involvement so that appropriate investigations are done on time and proper treatment instituted so that long term irreversible complications are prevented.

## References

[1] Arino H, Armangue T, Petit-Pedrol M, Sabater L, Martinez-Hernandez E, Hara M, Lancaster E (2016). Anti-LGIl-associated cognitive impairment: presentation and long-term outcome. Neurology.

[2] Irani SR, Alexander S, Waters P, Kleopa KA, Pettingill P, Zuliani L (2010). Antibodies to Kv1 potassium channel-complex proteins leucine-rich, glioma inactivated 1 protein and contactin-associated protein-2 in limbic encephalitis, Morvan’s syndrome and acquired neuromyotonia. Brain.

[3] van Sonderen A, Petit-Pedrol M, Dalmau J, Titulaer MJ (2017). The value of LGI1, Caspr2 and voltage-gated potassium channel antibodies in encephalitis. Nat Rev Neurol.

[4] de Bruijn M, van Sonderen A, van Coevorden-Hameete MH, Bastiaansen AEM, Schreurs MWJ, Rouhl RPW (2019). Evaluation of seizure treatment in anti-LGIl, anti-NMDAR, and anti-GABABR encephalitis. Neurology.

[5] Irani SR, Gelfand JM, Bettcher BM, Singhal NS, Geschwind MD (2014). Effect of rituximab in patients with leucine-rich, glioma-inactivated 1 antibody-associated encephalopathy. JAMA Neurol.

[6] Li W, Wu S, Meng Q, Zhang X, Guo Y, Cong L (2018). Clinical characteristics and short-term prognosis of LGI1 antibody encephalitis: a retrospective case study. BMC Neurol.

[7] Wang M, Cao X, Liu Q, Ma W, Guo X, Liu X (2017). Clinical features of limbic encephalitis with LGI1 antibody. Neuropsychiatr Dis Treat.

[8] Lai M, Huijbers MGM, Lancaster E, Graus F, Bataller L, Balice-Gordon R (2010). Investigation of LGI1 as the antigen in limbic encephalitis previously attributed to potassium channels: a case series. The Lancet Neurology.

[9] Simabukuro MM, Nobrega PR, Pitombeira M, Cavalcante WCP, Grativvol RS, Pinto LF (2016). The importance of recognizing faciobrachial dystonic seizures in rapidly progressive dementias. Dement Neuropsychol.

[10] Navarro V, Kas A, Apartis E, Chami L, Rogemond V, Levy P (2016). Motor cortex and hippocampus are the two main cortical targets in LGI1-antibody encephalitis. Brain.

[11] Blinder T, Lewerenz J (2019). Cerebrospinal fluid findings in patients with autoimmune encephalitis-a systematic analysis. Front Neurol.

[12] Irani SR, Stagg CJ, Schott JM, Rosenthal CR, Schneider SA, Pettingill P (2013). Faciobrachial dystonic seizures: the influence of immunotherapy on seizure control and prevention of cognitive impairment in a broadening phenotype. Brain.

[13] Irani SR, Michell AW, Lang B, Pettingill P, Waters P, Johnson MR (2011). Faciobrachial dystonic seizures precede Lgi1 antibody limbic encephalitis. Ann Neurol.

[14] Grover S, Kukreti R (2014). HLA alleles and hypersensitivity to carbamazepine: an updated systematic review with meta-analysis. Pharmacogenet Genomics.

[15] van Sonderen A, Roelen DL, Stoop JA, Verdijk RM, Haasnoot GW, Thijs RD (2017). Anti-LGI1 encephalitis is strongly associated with HLA-DR7 and HLA-DRB4. Ann Neurol.

